# Drug-induced liver injury due to avacopan improved by mycophenolate mofetil: A case report

**DOI:** 10.1097/MD.0000000000042121

**Published:** 2025-04-11

**Authors:** Ryo Yamashita, Yusuke Izumi, Keisuke Takane, Akiyoshi Kinoshita, Jun Hiramoto

**Affiliations:** a Department of General Medicine, The Jikei University School of Medicine, Daisan Hospital, Komae-si, Tokyo, Japan; b Department of General Medicine, The Jikei University School of Medicine, Daisan Hospital, Komae-si, Tokyo, Japan; c Department of General Medicine, The Jikei University School of Medicine, Daisan Hospital, Komae-si, Tokyo, Japan; d Department of Gastroenterology and Hepatology, The Jikei University School of Medicine, Daisan Hospital, Komae-si, Tokyo, Japan; e Department of General Medicine, The Jikei University School of Medicine, Daisan Hospital, Komae-si, Tokyo, Japan.

**Keywords:** antineutrophil cytoplasmic antibody-associated vasculitis, avacopan, drug-induced liver injury, mycophenolate mofetil

## Abstract

**Rationale:**

Antineutrophil cytoplasmic antibody–associated vasculitis (AAV) is a systemic necrotizing vasculitis that predominantly affects small vessels. Glucocorticoids are the standard therapeutic agents for AAV; however, their long-term use can cause damage. Avacopan is a small-molecule complement component 5a receptor antagonist that reduces vasculitis and can potentially be used as an alternative to glucocorticoids. However, its therapeutic efficacy remains unknown, and drug-induced liver injury (DILI) is a concern associated with its use.

**Patient concerns:**

A 74-year-old woman with a history of granulomatosis with polyangiitis was admitted to our hospital with a 1-week history of fatigue and anorexia.

**Diagnoses:**

She had started avacopan 3 months before hospitalization. Blood tests showed severe liver injury, and since other diseases were ruled out, she was diagnosed with DILI secondary to avacopan.

**Interventions:**

Avacopan was discontinued, and glucocorticoid doses were increased and ursodeoxycholic acid was administered; however, the liver injury did not resolve. Therefore, mycophenolate mofetil (MMF) was started.

**Outcomes:**

The liver injury was resolved after starting MMF.

**Lessons:**

MMF is effective in treating DILI caused by avacopan.

## 1. Introduction

Antineutrophil cytoplasmic antibody (ANCA)-associated vasculitis (AAV) is a systemic necrotizing vasculitis with few or no immune deposits, predominantly affecting small vessels, associated with myeloperoxidase (MPO)-ANCA or proteinase 3 (PR3)-ANCA.^[1]^ AAV may have life-threatening complications, including rapidly progressive glomerulonephritis and alveolar hemorrhage.^[2,3]^ Glucocorticoids are the mainstay of treatment for AAV, but long-term use can cause a variety of damage.^[4]^ The possibility of reducing glucocorticoid doses with the combination of rituximab has been reported, but glucocorticoids remain the mainstay of treatment.^[5]^ Avacopan is a small-molecule complement component 5a (C5a) receptor antagonist that selectively suppresses the effects of C5a. Inhibition of C5a receptors blocks chemoattraction and activation of neutrophils. This mechanism improves the disease activity of AAV. The ADVOCATE trial suggests that avacopan may be an alternative to glucocorticoids and may reduce glucocorticoid toxicity.^[6]^ A few cases of drug-induced liver injury (DILI) due to avacopan have been reported, but most cases improve with discontinuation of avacopan and use of ursodeoxycholic acid (UDCA).^[7]^ We herein report a case of DILI that did not improve after discontinuation of avacopan and administration of UDCA, but improved after administration of mycophenolate mofetil (MMF).

## 2. Case presentation

**Figure 1.**
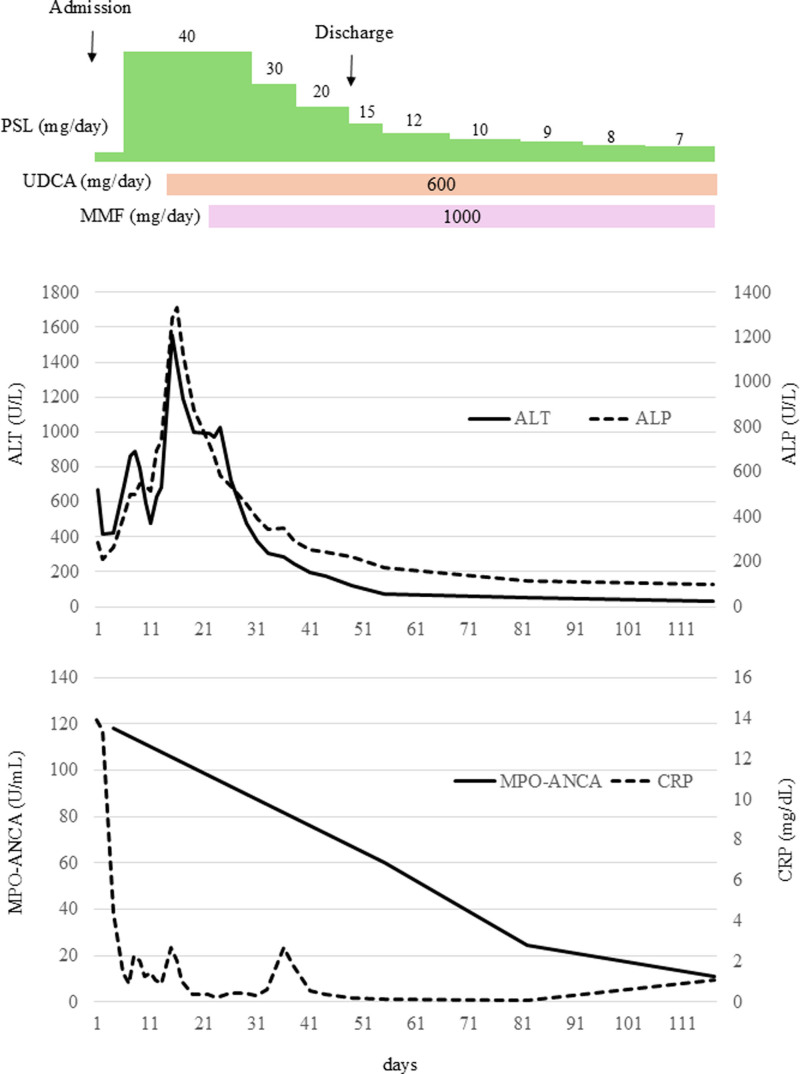
Clinical course of liver injury due to avacopan. ALP = alkaline phosphatase, ALT = alanine aminotransferase, CRP = C-reactive protein, MMF = mycophenolate mofetil, MPO-ANCA = myeloperoxidase antineutrophil cytoplasmic antibody, PSL = prednisolone, UDCA = ursodeoxycholic acid.

A 74-year-old woman was admitted to our hospital with a complaint of slowly progressive anorexia and fatigue. Five years prior to admission, she was admitted to our hospital with fever, multiple pulmonary nodules and renal failure. She was diagnosed with granulomatosis with polyangiitis (GPA) based on bronchoscopy findings of necrotizing granulomatous vasculitis and elevated serum MPO-ANCA. Her multiple pulmonary nodules and proteinuria disappeared following induction remission therapy with prednisolone (PSL) and 4 doses of weekly rituximab (500 mg/body). The dose of PSL was gradually reduced while checking the disease activity. Azathioprine was started as maintenance therapy during the course of the disease, but was discontinued because of thrombocytopenia. Finally, the patient was maintained on 3 mg/day of PSL without worsening disease activity. A persistent fever occurred 3 months prior to admission, and blood tests showed elevated C-reactive protein (CRP) and MPO-ANCA. Infection was ruled out and the patient was considered to be having a flare of GPA. PSL was increased to 30 mg/d and avacopan was initiated at 60 mg/d, resulting in a resolution of her fever. The CRP also became negative and so the MPO-ANCA titer was decreased. Two weeks prior to admission PSL was gradually reduced to 9 mg/day. A week before admission, she experienced fatigue and anorexia. She visited our hospital as her symptoms gradually worsened. No abnormalities were found on physical examination of her abdomen. Laboratory data showed no renal impairment and MPO-ANCA titers were lower compared to 2 weeks prior. In contrast, severe hepatic abnormalities were observed: aspartate aminotransferase (AST) 233 U/L, alanine aminotransferase (ALT) 670 U/L, lactate dehydrogenase (LDH) 302 U/L, total bilirubin (T-Bil) 4.0 mg/dL, alkaline phosphatase (ALP) 285 U/L and γ-glutamyl transpeptidase (γ-GTP) 595 U/L. CRP was only slightly elevated at 0.94 mg/dL. Albumin (Alb) was maintained at 3.7 g/dL. There was no anemia, thrombocytopenia, or coagulation abnormality. There was no alcohol consumption, new medications, or supplements, except avacopan, within 2 years. Whole body enhanced computed tomography (CT) showed no pulmonary nodules, no periportal edema, no thickening of the gallbladder wall, or any biliary obstruction. Abdominal ultrasound and magnetic resonance cholangiopancreatography also showed that the gallbladder and bile ducts were intact. Further blood test results were as follows: hepatitis A IgM negative, hepatitis B surface antigen negative, IgM antibody to hepatitis B core antigen negative, hepatitis C antibody negative, Epstein–Barr virus (EBV) viral capsid antigen (VCA) immunoglobulin-G (IgG) positive, EBV VCA-IgM negative, EBV nuclear antigen positive, cytomegalovirus (CMV) IgG positive, CMV IgM negative, antinuclear antibody negative, antiliver kidney microsome-1 antibody negative, antismooth muscle antibody negative and antimitochondrial M2 antibody negative. These results suggested that liver injury due to viral infection, autoimmune hepatitis and primary biliary cholangitis (PBC) was unlikely. The possibility of liver damage due to GPA was considered unlikely as CRP elevation was mild, MPO-ANCA titer tended to improve and renal damage and pulmonary nodules did not worsen. Therefore, DILI was highly suspected. Excepting avacopan, she was taking PSL, azilsartan, bisoprolol, sulfamethoxazole-trimethoprim (ST), edoxaban, esomeprazole and ramelteon at the time of admission and for more than 2 years. The patient’s Revised Electronic Causality Assessment Method score was 11, and in the definite/highly likely group; therefore, the possibility of DILI secondary to avacopan was high. We discontinued avacopan from the time of admission and monitored hepatobiliary enzymes. Despite the discontinuation of avacopan, hepatobiliary enzymes gradually increased to AST 681 U/L, ALT 890 U/L, T-Bil 11.4 mg/dL, ALP 499 U/L, and γ-GTP 702 U/L by day 8. PT was in the normal range, but Alb dropped to 2.5 g/dL. We increased PSL to 40 mg/d for DILI. After the PSL dose was increased, hepatobiliary enzymes showed a temporary tendency to improve, but worsened again, with AST 937 U/L, ALT 1557 U/L, T-Bil 28.8 mg/dL, ALP 1282 U/L, and γ-GTP 2087 U/L on day 15. As DILI worsened during glucocorticoid administration, we added UDCA. After initiating UDCA, the liver injury improved to AST 308 U/L, ALT 999 U/L, T-Bil 21.8 mg/dL, ALP 876 U/L, and γ-GTP 1626 U/L on day 19. Although T-bil and ALP steadily improved thereafter, a re-elevation of liver enzymes was conducted on day 24, with AST 420 U/L and ALT 1028U/L. As CRP was not elevated and CMV antigemia was negative, hepatotoxicity associated with infection was considered negative. Because she had DILI refractory to treatment, acute liver failure resulted and MMF 1000 mg/d was commenced on day 24. After MMF initiation, liver injury gradually improved, improving liver enzymes to AST 81 U/L, ALT 372 U/L, T-Bil 5.6 mg/dL, ALP 395 U/L, and γ-GTP 925 U/L by day 31. Alb also improved to 3.0 g/dL. We therefore reduced the PSL dose to 30 mg/day. PSL was subsequently reduced again and she was discharged on day 45 without recurrence of liver injury. Further PSL reduction was performed on an outpatient basis, and the liver injury did not recur (Fig. 1). The Birmingham Vasculitis Activity Score was 0, CRP was negative, and MPO-ANCA was improving, so we considered that there was no GPA relapse. There was concern about liver damage due to cytomegalovirus antigenemia, therefore the patient was followed several times, but remained negative.

## 3. Discussion

The avacopan group had lower disease activity at 52 weeks compared to the glucocorticoid group in the ADVOCATE trial. Glucocorticoid-induced adverse events were also lower in the avacopan group. In this study, 9 of 166 patients treated with avacopan showed liver injury, which resolved after avacopan withdrawal.^[6]^ Liver injury was observed in 3 of 107 patients treated with avacopan and rituximab.^[8]^ Although no statistical analysis was performed, the frequency of liver injury was not significantly different between the avacopan and glucocorticoid groups.

DILI is typically classified as direct or idiosyncratic. Direct hepatotoxicity is caused by drugs that are inherently toxic to the liver, while idiosyncratic hepatotoxicity is caused by drugs that have little intrinsic toxicity and only rarely cause liver damage.^[9]^ Idiosyncratic hepatotoxicity is classified as hepatocellular, cholestatic or mixed according to the *R* value calculated from the alanine aminotransferase and alkaline phosphatase values. The *R* value is the ratio of alanine aminotransferase to the upper reference limit divided by the ratio of alkaline phosphatase to the upper reference limit. If the *R* value is 5 or higher, the liver injury is classified as hepatocellular, but if the *R* value is 2 or lower, the liver injury is classified as cholestatic. If the *R* value is between 2 and 5, the liver injury is classified as mixed.^[10]^ The present case is classified as hepatocellular liver injury given an *R* value of 11.5. Hepatocellular liver injury occurs within days to months after administration of the causative agent.^[9]^ Since the only drug initiated during this period was avacopan, it was the most suspected causative agent for DILI. ST is also a typical cause of DILI. However, ST is unlikely to be the causative agent of DILI in this case. This is because 4 years had already passed since the patient started ST and no liver injury had occurred after the patient resumed ST. Although ST often interacts with other drugs and lead to DILI, the patient was not concomitantly treated with drugs other than avacopan during the 2 years prior to admission. Moreover, patients with renal impairment are prone to adverse events, including DILIs; however, the patient did not develop dysfunction in other organs, such as renal dysfunction, before admission. These findings also suggested that ST was not a potential cause of DILI in the present case.

Hepatic injury has been reported to correlate with disease activity in AAV and liver injury may occur when disease activity is high. It has been reported that liver injury is more common with GPA than with microscopic polyangiitis (MPA).^[11]^ There were no other findings suggestive of AAV-related symptoms in this case. MPO-ANCA titers also tended to decrease with the use of glucocorticoids. Therefore, we considered the possibility of liver damage associated with AAV to be low. A few cases of PBC complicating AAV have been reported.^[12]^ Antimitochondrial antibodies were negative in this case, making the possibility of PBC unlikely. Although the observed efficacy of MMF suggested IgG4-related sclerosing cholangitis,^[13]^ magnetic resonance cholangiopancreatography did not support this diagnosis.

Withdrawal of the offending drug is the primary treatment for DILI. Glucocorticoids may be considered if the liver injury worsens progressively after drug withdrawal.^[14]^ Although there is no established evidence, UDCA may be effective in patients with cholestatic liver injury and pruritus.^[15]^ Several cases of DILI due to avacopan that improved with UDCA have been reported.^[7,16,17]^ In these reports, as in this case, AST, ALT, ALP, and T-Bil values were persistent after the discontinuation of avacopan; however, it promptly improved after initiating UDCA. By contrast, in the present case, UDCA was initiated in addition to discontinuing avacopan and increasing the dose of glucocorticoids; however, the ALT values did not improve immediately. The discontinuation of UDCA was considered based on its limited effect and the *R* values raising the suspicion of DILI. However, the mechanism of avacopan-induced DILI remains unknown, and several studies reported patients experiencing improvement with UDCA treatment alone.^[7,16,17]^ We continued the treatment with UDCA considering that avacopan might have induced DILI because to a combination of hepatocellular and cholestatic liver injury. In DILI with poor therapeutic response to glucocorticoids and UDCA, the efficacy of MMF has been reported, especially in cases when immune checkpoint inhibitors are used.^[18,19]^ The mechanisms of DILI owing to avacopan have not yet been elucidated. Considering that glucocorticoids were partially effective, conceivably, the metabolites of abacopan could have acted as haptens and caused liver damage through an allergic mechanism. In addition, the metabolites may have induced autoantibodies, leading to a condition similar to autoimmune hepatitis.^[20]^ MMF is a prodrug and is rapidly degraded in vivo to mycophenolic acid, which exerts its effect. The mechanism of action of mycophenolic acid is the inhibition of the purine synthesis pathway. Of the 2 purine synthesis pathways, denovo and salvage, mycophenolic acid inhibits inosine monophosphate dehydrogenase, the rate-limiting enzyme in the denovo pathway. Therefore, DNA synthesis is inhibited. Although many cells conduct DNA synthesis by the denovo and salvage pathways, in lymphocytes it is mainly conducted by the denovo pathway. Therefore, mycophenolic acid selectively suppresses lymphocyte proliferation and exerts an immunosuppressive effect.^[21]^

This immunosuppressive effect may have improved liver damage owing to allergic mechanisms or autoimmunity.

Liver biopsy should be considered to exclude other diseases including autoimmune hepatitis.^[22]^ However, in this case, the patient was taking direct oral anticoagulant because she had an implanted inferior vena cava filter for deep vein thrombosis; therefore, liver biopsy was not performed because of the high risk of bleeding and lack of patient consent.

In conclusion, this case suggests that MMF may be effective in treating liver injury caused by avacopan and that is refractory to treatment involving discontinuation of avacopan plus initiation of glucocorticoids and UDCA. To the best of our knowledge, this is the first report of MMF efficacy suggested for DILI caused by avacopan. Further evidence is needed regarding the efficacy of MMF in treating avacopan-induced liver injury.

## Acknowledgments

We thank Enago (www.enago.jp) for English language editing.

## Author contributions

**Conceptualization:** Ryo Yamashita, Akiyoshi Kinoshita.

**Investigation:** Ryo Yamashita.

**Project administration:** Ryo Yamashita.

**Supervision:** Jun Hiramoto.

**Writing – riginal draft:** Ryo Yamashita.

**Writing – review & editing:** Yusuke Izumi, Keisuke Takane, Akiyoshi Kinoshita, Jun Hiramoto.
